# A Protein Data Bank Survey Reveals Shortening of Intermolecular Hydrogen Bonds in Ligand-Protein Complexes When a Halogenated Ligand Is an H-Bond Donor

**DOI:** 10.1371/journal.pone.0099984

**Published:** 2014-06-16

**Authors:** Jarosław Poznański, Anna Poznańska, David Shugar

**Affiliations:** 1 Biophysics Department, Institute of Biochemistry and Biophysics PAS, Warszawa, Poland; 2 Centre for Monitoring and Analyses of Population Health Status, National Institute of Public Health - National Institute of Hygiene, Warszawa, Poland; 3 Institute of Biochemistry and Biophysics PAS, Warszawa, Poland; UMR-S665, INSERM, Université Paris Diderot, INTS, France

## Abstract

Halogen bonding in ligand-protein complexes is currently widely exploited, e.g. in drug design or supramolecular chemistry. But little attention has been directed to other effects that may result from replacement of a hydrogen by a strongly electronegative halogen. Analysis of almost 30000 hydrogen bonds between protein and ligand demonstrates that the length of a hydrogen bond depends on the type of donor-acceptor pair. Interestingly, lengths of hydrogen bonds between a protein and a halogenated ligand are visibly shorter than those estimated for the same family of proteins in complexes with non-halogenated ligands. Taking into account the effect of halogenation on hydrogen bonding is thus important when evaluating structural and/or energetic parameters of ligand-protein complexes. All these observations are consistent with the concept that halogenation increases the acidity of the proximal amino/imino/hydroxyl groups and thus makes them better, i.e. stronger, H-bond donors.

## Introduction

Specific non-covalent interactions of low-mass ligands with proteins drive properties of the enzymatic machinery in a living cell. According to (induced) key-lock theory (see [1] for review) a low-mass ligand should fit to a dedicated binding site that is accessible on the protein surface. This steric compatibility, known as van der Waals interactions, dominates ligand-protein selectivity, simply excluding the majority of putative ligands and favoring these that fit to the protein binding site. Other types of interactions modulate the stability of ligand-protein complexes. The strongest ones are electrostatic interactions between charged groups (known as salt-bridges, formally zero momentum in multipole expansion of electrostatic interactions), which energy frequently exceeds 10 kcal/mol.

Hydrogen bonding is the next type of interactions proven to contribute significantly to stabilization of protein structure and to the organization of intermolecular complexes (ca. 3 to 5 kcal/mol). The energy of a single hydrogen bond (H-bond) in ligand-protein complexes depends both on the type of hydrogen bond donor (D) and acceptor (A) and on the overall geometry of the D-H•••A system. The shortest H-bonds are observed for oxygen acting as a donor, OH•••O (2.70Å) and OH•••N (2.88Å), respectively. When nitrogen is an H-bond donor, its distance to an acceptor is longer: NH•••O (3.04Å) and NH•••N (3.10Å), respectively [2]. Subsequently, numerous non-canonical weak H-bonds have been identified by statistical analyses of protein structures, and previously identified in crystals of low-mass compounds. This includes, amongst others, a π electron system acting as an H-bond acceptor [3]–[5], and an aliphatic carbon acting as an H-bond donor [4], [6], [7].

During the last decade, halogen bonding (X-bond, see [8] for review) has been recognized to play a similar role as H-bonding in protein-ligand complexes. Halogen bonds have been identified in many crystal structures of low-mass compounds and their supramolecular ensembles [8]–[17], as well as in complexes of biomolecules with halogenated ligands [18]–[20]. Bearing in mind that numerous natural drugs, and an increasing number of synthetic drug candidates, are halogenated [21]–[23]; understanding the nature and thermodynamics of halogen bonding should contribute to rational drug design. Currently, halogenated compounds are widely used in screening libraries, and comprise almost 20% of low-mass protein ligands listed in the Protein Data Bank (PDB). The role of halogenated ligands in biological systems has been widely reviewed, amongst others, by Auffinger *et al.* [18], Parisini *et al.* [24], Rendine *et al.*, Voth & Ho [25], Voth *et al.* [26], Wilcken *et al.* [27] and Poznański & Shugar [28].

However, there is some controversy about the energy of a halogen bond. In aqueous medium estimates of intra- or intermolecular halogen bonds vary from 0.2 [29] up to 5–8 kcal/mol [25], suggesting that, in biological systems, halogen- and hydrogen bonds may be of similar strength. However, the apparently largest values for an X-bond were obtained *ab initio* for CF_3_-X•••NH_3_ systems: 2.3, 4.7 and 6.4 kcal/mol for X = Cl, Br and I, respectively [30]. These values agree with energies estimated by IR spectroscopy, for CF_3_-X•••N(CH_3_)_3_ in liquid noble gases, which are the best models for a non-polar solvent that does not interfere with solute-solute interactions: 2.1, 4.4 and 6.8 kcal/mol for X-bonds involving Cl, Br and I, respectively [31]–[33]. These halogen bonds can compete with hydrogen bonding, as well documented for numerous low-mass complexes *in silico* [34], [35], in solution [36], [37], and in the solid state [17], [38], [39].

Due to this revived interest in halogen bonding, the observed effect of a halogen atom on structural stability [25], [40], or ligand binding [41], [42], has been attributed to a direct effect of halogen bonding only. However, the strong electronegative and hydrophobic character of halogen atoms may also contribute to intra- and intermolecular interactions. For example we have recently shown that inhibitory activities (IC_50_) against protein kinase CK2α observed for a series of benzotriazoles brominated on the benzene ring can be explained by a balance of hydrophobic and electrostatic interactions [43].

Halogenation modulates electron density on proximal donors and acceptors of hydrogen bonds [44], as well as changes in protonation equilibria of proximal dissociable groups [45], [46]. Well-known examples include the decrease in pK_a_ of fluorinated alcohols [47]: ethanol vs. 2,2′,2″-trifluoroethanol (pK_a_ decrease by 3.45) and phenol vs. pentafluorophenol (pK_a_ decrease by 4.4).

Likewise, halogenation of uracil was shown to reduce the hydrogen-bond-accepting, and to increase the hydrogen-bond-donating, capabilities of halogenated DNA bases [48]–[50]. Other illustrative examples of the direct effect of a halogen atom on strengthening of proximal hydrogen bonds are brominated natural [51], [52] and synthetic [53]–[55] DNA, which were found to be much more stable than the corresponding non-brominated analogues.

A further example of the foregoing is the report of Xu *et al.* [42] on a series of closely related halogenated inhibitors of phosphodiesterase 5 (PDE5). There are five PDB structures of PDE5 with bound inhibitors that differ only by substitution of a hydrogen atom by F, Cl, Br or I, respectively (see PDB entries 3TSE, 3SHY, 3SHZ, 3SIE, 3TSF). Location of these closely related ligands in the binding pocket was judged to be stabilized, besides two hydrogen bonds and numerous vdW interactions, by intermolecular interaction between the halogen atom (X) and the hydroxyl oxygen of Tyr612. However, there are also two intermolecular hydrogen bonds between the side-chain of Gln817, and ligands Od and Ns, respectively, both proximal to the halogen atom (3 chemical bonds distance). Changes in the lengths of these, upon variation of the halogen substituent, reflects eventual strengthening of these H-bonds, not taken into account by the authors [42].

To our knowledge, no high-throughput analyses addressing the effect of a halogen atom on proximal hydrogen bond(s) have yet been reported for ligand-protein systems [13], [19], [24], [25], [56]–[61]. We herein analyze the effect of the halogen atom of a halogenated ligand on the lengths of hydrogen bonds (both proximal and distal), identified in two families of proteins: protein kinases (EC 2.7) and acyltransferases (EC 2.3).

## Results and Discussion

### 1 PDB Screening

To avoid the eventual effect of protein-specific ligand binding modes, two protein families were analyzed. Protein kinases (EC 2.7) and acyltransferases (EC 2.3) are the proteins for which the largest number of structures with halogenated ligands was identified in the PDB. All complexes of ligands with proteins of these two families were analyzed. A total of 3852 PDB entries was found, 3187 with non-halogenated ligands, LH, 505 with fluorinated ones, LF, and 408 containing halogenated (not fluorinated) ligands, LX, contributing together to 1228 records of acyltransferases and 2624 records of protein kinases. After exclusion of protein sulfur as either hydrogen bond acceptor or donor, a total number of 24470 hydrogen bonds was identified, 1930 with fluorinated, 1390 with halogenated ligands, and 21150 with non-halogenated ligands, respectively. In addition, 41 intermolecular H-bonds to protein sulfur (Met or Cys) were excluded from further analyses (see Table 1 for the short statistics).

**Table 1:** Occurrence of various types of hydrogen bonds identified in two groups of proteins (Enzyme Classification, EC, 2.3 or 2.7) for three types of ligands.

Ligand contribution to H-bond	Ligand type								
	Non-halogenated (LH)			Fluorinated (LF)			Halogenated but not fluorinated (LX)		
	EC		Total	EC		Total	EC		Total
	2.3	2.7		2.3	2.7		2.3	2.7	
N - acceptor	87	1764	1851	23	339	362	6	294	300
N - donor	2737	2939	5676	355	490	845	237	424	661
O - acceptor	3329	6708	10037	266	344	610	124	218	342
O - donor	1112	2510	3622	82	34	116	36	53	89
Total	7265	13921	21186	726	1207	1933	403	989	1392

These data include 41 hydrogen bonds to protein sulfur, which were excluded from further analyses.

### 2 Distribution of Hydrogen Bond Lengths as a Function of H-bond Topology

**Figure 1:**
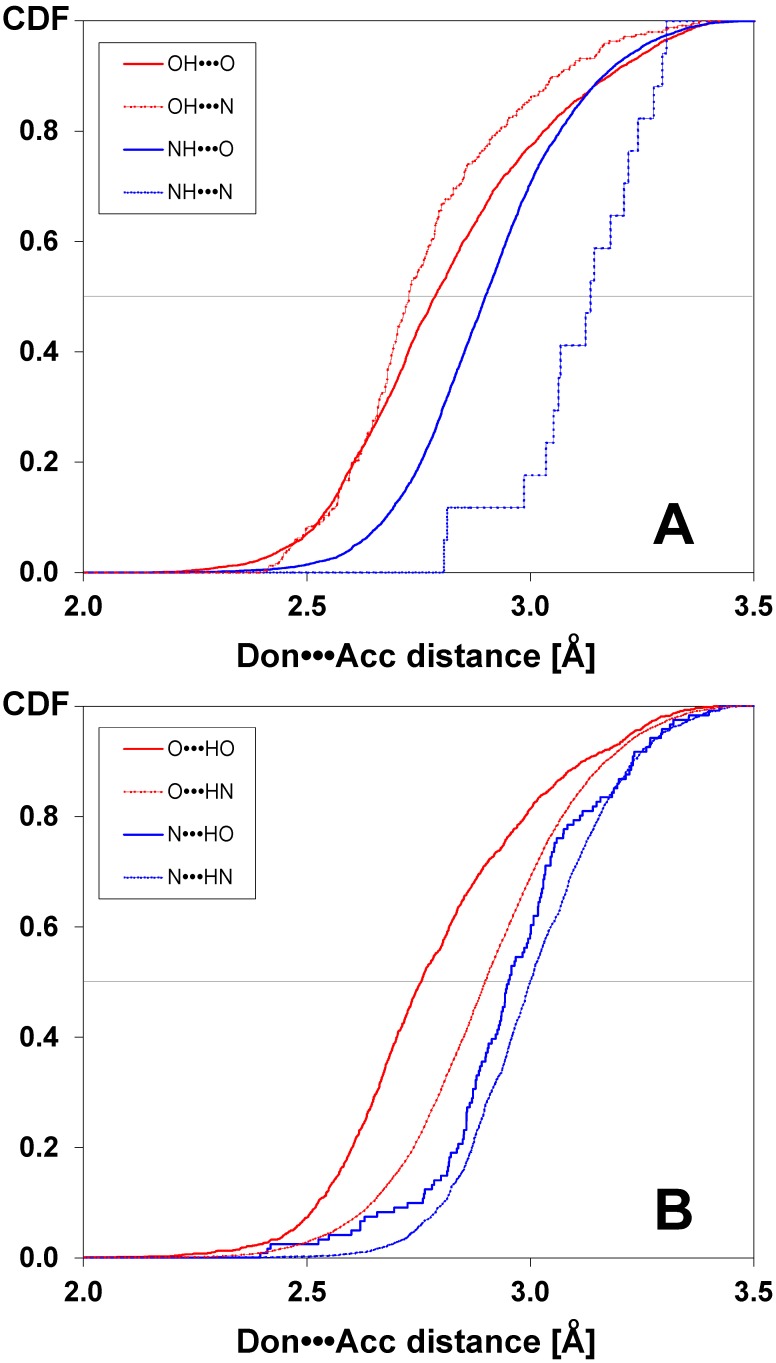
Cumulative distributions of donor-acceptor distances determined for various types of intermolecular hydrogen bond donor-acceptor pairs identified in complexes of proteins with non-halogenated ligands, in which the ligand is either a hydrogen bond donor (A) or acceptor (B).

Hydrogen bonds were grouped according to eight possible topologies of hydrogen bond donor-acceptor pairs, i.e. N-H_lig_•••O_prot_ (NH•••O), N_lig_•••H-O_prot_ (N•••HO), O-H_lig_•••O_prot_ (OH•••O), O_lig_•••H-O_prot_ (O•••HO), N-H_lig_•••N_prot_ (NH•••N), N_lig_•••H-N_prot_ (N•••HN), O-H_lig_•••N_prot_ (OH•••N), and O_lig_•••H-N_prot_ (O•••HN). The distributions obtained for non-halogenated ligands are presented in Figure 1. For ligands acting as H-bond donors, two left shifted distributions identify the topologies of the shortest H-bonds types (i.e. OH•••N and OH•••O), medians of which are the lowest (Figure 1A). The intermediate distribution (NH•••O) is also characterized by a higher median. The last one (NH•••N) is much more shifted to the right, and its median is the highest. The order of distributions strictly correlates with the average strength of H-bonds: the shorter is donor-to-acceptor distance, the stronger is the H-bond. This agrees with a common order of an average enthalpy of formation of various types of hydrogen bonds in biomolecules: OH•••N, 6.9 kcal/mol; OH•••O, 5.0 kcal/mol; NH•••N, 3.1 kcal/mol, and NH•••O, 1.9 kcal/mol, respectively [62].

Inspection of cumulative distributions for non-halogenated ligands acting as H-bond acceptors (see Figure 1B) clearly demonstrated that hydrogen bonds involving two oxygen atoms are statistically the shortest, as evidenced by the left-shift of the cumulative distribution function towards shorter distances (and also smaller medians). H-bonds between two nitrogen atoms are the longest, and the two remaining types of hydrogen bonds of mixed topology, N•••HO and O•••HN are intermediate in their lengths.

Formal statistical analysis clearly demonstrated that most topologies of an H-bond differ significantly according to the H-bond donor-to-acceptor distance distribution. Interestingly, these differences are observed even between pairs in which the proton is swapped between the ligand and the protein. Hence, the distribution of OH•••O (OH_lig_•••O_prot_) differs from that of an O•••HO (O_lig_•••HO_prot_), p<10^−2^. Similarly OH•••N differs from O•••HN (p<·10^−10^) and NH•••O differs from N•••HO (p = 0.05). This significant asymmetry may be a consequence of overrepresentation of several types of H-bond donors and acceptors in proteins. Namely, oxygen acceptors are dominated by backbone carbonyls, oxygen donors are Ser, Thr and Tyr hydroxyl groups, nitrogen donors are mainly backbone amides, and nitrogen donors are solely the imidazole of His, which are rare in proteins. In consequence, the protein nitrogen H-bond acceptors are strongly underrepresented (see column ‘n’ in Table 2).

**Table 2:** Results of the Kruskal-Wallis (K-W) test in the analysis of the topology-dependent length of a hydrogen bond between a non-halogenated ligand (LH) and a protein: for each pair of hydrogen bond acceptor/donor pair the p-value for the null hypothesis that both distributions are identical was estimated according to the two-tailed multiple comparison.

H-bond topology	n	Mean rank	H-bond topology (ligand • protein)							
			OH•••O	OH•••N	NH•••O	NH•••N	O•••HO	O•••HN	N•••HO	N•••HN
**LH: K-W test for non-halogenated ligands: H(7, N = 21150) = 1519; p<10^−9^**										
**OH•••O**	**3358**	**8378**	-	**1.9·10^−3^**	**<1·10^−10^**	**1.6·10^−7^**	**7.6·10^−3^**	**<1·10^−10^**	**<1·10^−10^**	**<1·10^−10^**
OH•••N	251	6787	**1.9·10^−3^**	-	**<1·10^−10^**	**6.2·10^−10^**	1	**<1·10^−10^**	**<1·10^−10^**	**<1·10^−10^**
**NH•••O**	**5670**	**11015**	**<1·10^−10^**	**<1·10^−10^**	-	**1.4·10^−3^**	**<1·10^−10^**	1	**0.05**	**<1·10^−10^**
NH•••N	17	17026	**1.6·10^−7^**	**6.2·10^−10^**	**1.4·10^−3^**	-	**9.1·10^−9^**	**1.1·10^−3^**	0.20	1
O•••HO	1331	7659	**7.6·10^−3^**	1	**<1·10^−10^**	**9.1·10^−9^**	-	**<1·10^−10^**	**<1·10^−10^**	**<1·10^−10^**
**O•••HN**	**8675**	**10943**	**<1·10^−10^**	**<1·10^−10^**	1	**1.1·10^−3^**	**<1·10^−10^**	-	**0.03**	**<1·10^−10^**
N•••HO	121	12769	**<1·10^−10^**	**<1·10^−10^**	**0.05**	0.20	**<1·10^−10^**	**0.03**	-	0.47
**N•••HN**	**1727**	**14140**	**<1·10^−10^**	**<1·10^−10^**	**<1·10^−10^**	1	**<1·10^−10^**	**<1·10^−10^**	0.47	-

The values marked in red denote the pairs of distributions that differ one from the other, with α = 0.05. Additionally, the identified number of each type of hydrogen bond, n, and mean rank test are presented.

The foregoing is valid for all types of ligands acting as either acceptor or donor of an H-bond (see Figure S1 and Table S1). The statistical significance of the observed differences in donor-acceptor distance distributions was evaluated, separately for the three types of ligands, with the aid of the non-parametric Kruskal-Wallis test (p<10**^−^** ^9^). Since these differences were found globally significant, the post-hoc approach was used to identify those pairs that significantly differ. Estimated p-values, together with the number of identified H-bonds, and mean rank of donor-acceptor distances, are presented in Table 2 and Table S1.

The majority of the analyzed pairs of distributions for non-halogenated ligands (LH) differ significantly (23 out of 28, assuming a significance level of 0.05). In the case of fluorinated (LF) and other halogenated ligands (LX), the small number of identified hydrogen bonds of the type N•••HO (N_lig_•••H-O_prot_ with n = 4 or 2 H-bonds found for LH and LX ligands, respectively) and NH•••N (NH_lig_•••N_prot_ with n = 1 and 0, respectively), precluded analysis of these two types of hydrogen bonds. For the remaining groups, distributions for 11 out of 14 possible pairs differ significantly both for fluorinated (LF) and otherwise halogenated (LX) ligands (Table S1). In this context, the hydrogen bond lengths to halogenated or non-halogenated ligands must be compared separately for eight groups representing all possible topologies of hydrogen bonding in ligand-protein complexes. Otherwise, the differences in representation of various types of hydrogen bonds would contribute in an uncontrolled manner to the observed distance distributions.

**Figure 2:**
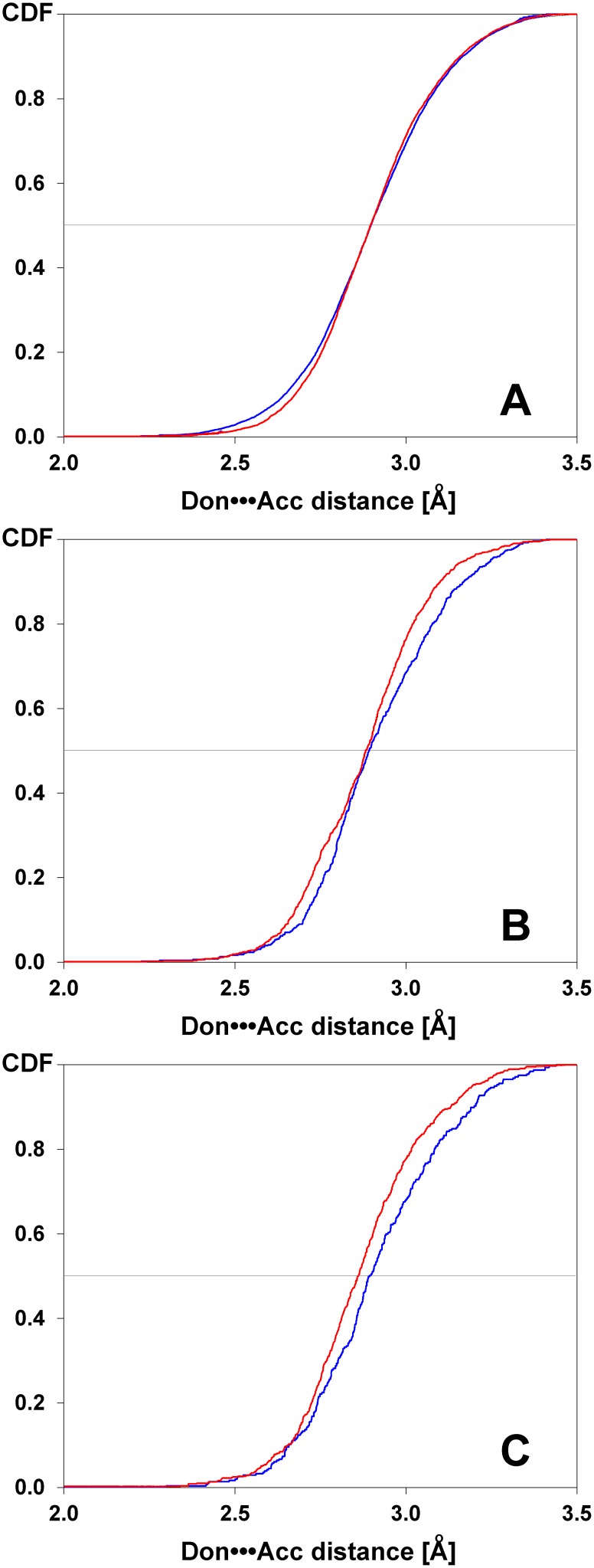
Cumulative distributions of NH•••O (blue) and O•••HN (red) intermolecular hydrogen bonds identified in protein complexes with non-halogenated (A, LH), fluorinated (B, LF) and otherwise halogenated ligands (C, LX). See Table 2 for details.

The most common types of ligand-protein intermolecular hydrogen bonds in the PDB, NH•••O and O•••HN, display almost identical distributions for non-halogenated ligands (LH, see Figure 2A), but become visibly different for fluorinated (LF) or otherwise halogenated (LX) ligands (see Figure 2B, C). In both latter cases the distributions of the NH•••O hydrogen bond lengths are shifted left relative to those of the O•••HN. However, for fluorinated ligands the medians are, by chance, almost equal. The observed differences are statistically significant only for halogenated ligands (p = 0.03), but they are also probable for fluorinated ligands (p = 0.09) (see Table 2). It should be stressed that the observed differences in medians for fluorinated (LF) and halogenated (LX) ligands (0.01 and 0.03 Å respectively, see Table 3) exceed the precision of PDB records. Overall, this clearly shows that ligand substitution with electronegative atoms (F, Cl, Br, I) results in variation of the lengths of intermolecular hydrogen bonds. Moreover, this effect strongly depends on the type of hydrogen bond (Table S1).

**Table 3:** Comparison of distributions of hydrogen bond lengths, calculated separately for fluorinated (LF), otherwise halogenated (LX), and non-halogenated ligands (LH), for the four most represented topologies of protein-ligand hydrogen bonds.

H-bond topology	n		Mean rank			U statistics	Z_U_	p-value	Median [Å]			p-value
	LH	LF	LH		LF				LH	LF	Δ(LF−LH)	
**OH•••O**	3358	103	1743.9	1309.5		129525	**4.35**	**1.4·10^−5^**	**2.787**	**2.668**	**−0.119**	**7.2·10^−6^**
**NH•••O**	5670	842	3286.9	3051.9		2214771	**3.38**	**7.1·10^−4^**	2.899	2.885	**−**0.014	0.18
O•••HN	8675	571	4616.4	4731.6		2414967	**−**1.00	0.32	2.900	2.895	**−**0.005	0.76
**N•••HN**	1727	357	1060.4	956.0		277400	**2.98**	**2.9·10^−3^**	**3.001**	**2.976**	**−0.025**	**0.03**
	**LH**	**LX**	**LH**	**LX**					**LH**	**LX**	**Δ(LX−LH)**	
OH•••O	3358	72	1717.0	1645.5		115847	0.61	0.54	2.787	2.769	**−**0.018	0.35
**NH•••O**	5670	659	3201.7	2849.2		1660152	**4.69**	**2.8·10^−6^**	**2.899**	**2.864**	**−0.035**	**4.9·10^−5^**
O•••HN	8675	317	4492.7	4601.6		1341680	**−**0.73	0.46	2.900	2.897	**−**0.003	0.69
N•••HN	1727	298	1014.2	1006.0		255250	0.22	0.82	3.001	2.998	**−**0.003	0.81

Those for which hydrogen bonds to LX/LF ligands are, according to the Mann-Whitney U test, significantly shorter (assuming α = 0.05) are highlighted. Note that for each pair of H-bond distributions, a smaller mean rank indicates statistically shorter donor-acceptor distances, or, equivalently, positive values of Z_U_ statistics indicate these types of H-bonds, which are shorter to halogenated ligands. The corresponding medians, and their differences with statistical significances (p), are also presented.

### 3 Hydrogen Bonding to Halogenated or Fluorinated vs. Non-halogenated Ligands

**Figure 3:**
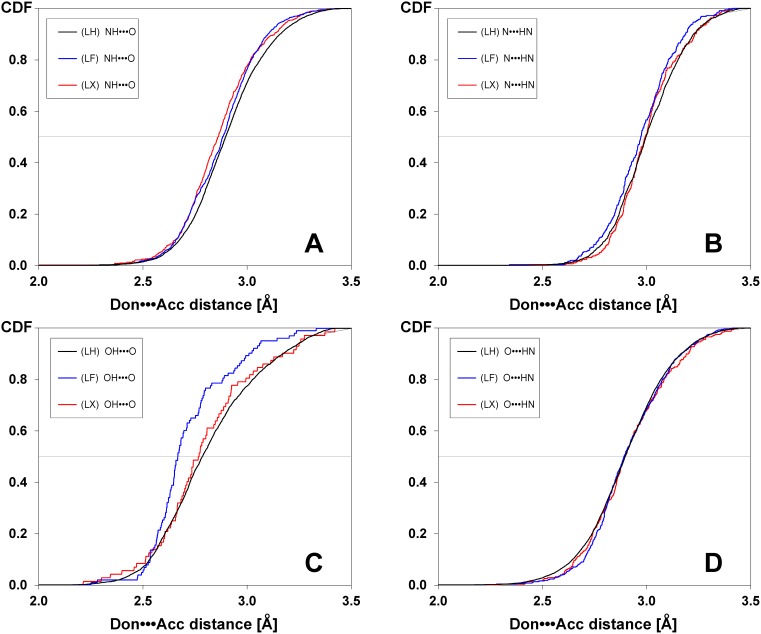
Effect of a halogen atom on cumulative distributions determined for the four most abundant types of hydrogen bond donor-acceptor pairs: NH•••O (A), OH•••O (B), N•••HN (C), and O•••HN (D), respectively. The distributions estimated for non-halogenated (LH), fluorinated (LF) and other halogenated ligands (LX) are presented in black, blue and green, respectively. See Table 3 for details.

The effect of a halogen atom on the distribution of hydrogen bond lengths was analyzed separately for the four most abundant types of hydrogen bonds: OH•••O, NH•••O, N•••HN and O•••HN (i.e. a protein oxygen being a hydrogen bond acceptor and a protein nitrogen being a hydrogen bond donor, see Table 3 for numbers). Cumulative distributions of hydrogen bond lengths estimated for fluorinated (LF) and otherwise halogenated (LX) ligands are, for some of the H-bond topologies, shifted towards shorter distances in comparison to non-halogenated ligands (LH). It is shown in Figure 3, and also confirmed by lower mean ranks collected in Table 3. Substitution with halogen atom mostly affects the lengths of OH•••O hydrogen bonds (Figure 3C). Smaller, but still visible, changes are observed for NH•••O (Figure 3A) and N•••HN (Figure 3B), while almost no variations are observed for O•••HN hydrogen bonds (Figure 3D). This is fully confirmed by the Mann-Whitney U test (see Table 3, and Table S2 for all H-bond topologies). Amongst them, hydrogen bonds to fluorinated ligands (LF) are significantly shorter for five out of seven tested pairs of distributions, while halogenated ligands (LX) differ significantly from non-halogenated ones (LH) only for the NH•••O type. It should be stressed that the medians for H-bond length with halogenated ligands (either LF or LX) are generally lower than those for non-halogenated ones (LH). This can also be easily checked via mean ranks (LX<LH and LF<LH).

In general the effect of fluorine vs. other halogens atoms follows the electronegativity scale. Fluorine changes properties of nitrogen both as acceptor and donor of hydrogen, and oxygen as donor of a hydrogen bond, whereas chlorine, bromine and iodine affect only hydrogen bond donors (both oxygen and nitrogen). The latter effect is clearly detectable for medians (decrease by 0.06 and 0.03 Å for OH•••O and NH•••O, respectively) and is statistically significant (p = 0.02 and p<10^−5^, see Table 3 for details). No variations are observed for halogenated ligands (LX) acting as H-bond acceptor. It is worth noting that the observed differences agree with *ab initio* simulations of base pairing of halogenated uracil with adenine [48].

### 4 Validation of the Differences Observed for Distributions of H-bond Donor-acceptor Distance

Proteins form H-bonds *via* various types of donors and acceptors. In view of the adopted approach, all types of hydrogen bonds should be analyzed separately, but the number of actually identified interactions with halogenated ligands makes results of such detailed analysis statistically insignificant. However, the large subset of H-bonds between ligands and protein backbone (i.e. carbonyl oxygen and amide nitrogen) enables analysis of much more homogenous subsets of protein-ligand interactions. The results generally agree with those obtained for all protein H-bond acceptors and donors (see Figure S2), confirming again the statistical significance of the effect of a halogen atom on lengths of intermolecular hydrogen bonds between a halogenated ligand and a protein.

A key point of the presented analysis is the significance of the results presented in the context of the quality of PDB structures. In fact, there are only a very limited number of X-ray structures of closely related halogenated ligands bound to the same protein that can be compared directly (e.g. PDE5 mentioned in the Introduction). Moreover, the resolution of X-ray structures precludes any direct interpretation of distances that differ by an order of 0.01 Å. All donor-acceptor distances must be regarded biased, but differences between observed distributions, as presented in Figures 1–3, may be considered as significant, since there is no factor explaining any systematic differences in biases for halogenated and non-halogenated ligands. However, to assess an eventual effect of quality of structures on the significance of observed differences in distance distributions, the analyses were repeated for two subsets of high-resolution X-ray structures, resolutions of which were better than 2.0 and 1.5 Å, respectively, and the general tendency to strengthening of H-bonds between protein and halogenated ligands (both LF and LX) acting as hydrogen bond donor was preserved (see Table S3).

## Materials and Methods

### Structural Data

The Protein Data Bank (PDB, [63]) was searched to identify all entries of protein kinases (EC 2.7) and acyltransferases (EC 2.3). Those containing ligands with at least one oxygen/nitrogen bound to a carbon atom were subjected to further analysis.

### Structural Analysis

All analyses were performed with the aid of the Yasara Model package [64]. For each class of protein, all intermolecular ligand-protein hydrogen bonds were identified, using 3.5 Å as a threshold for the distance between putative hydrogen bond donor and acceptor. The distributions of donor-acceptor distances were determined separately for three classes of ligands: non-halogenated (LH), fluorinated (LF) and others that are halogenated, but not fluorinated (LX). These data were then assigned to one of eight groups, according to the topology of the hydrogen bond. The latter was defined according to the ligand atom (oxygen or nitrogen) being either donor or acceptor of a hydrogen bond with protein nitrogen or oxygen. Since fluorinated ligands (LF) are the internal reference for the effect of other halogen atoms that may contribute in halogen bonding (LX), all heterogenic ligands, which were simultaneously fluorinated and modified with chlorine/bromine/iodine, were excluded from the analysis.

The most abundant types of hydrogen bonds (i.e. NH_lig_•••O_prot_, OH_lig_•••O_prot_, O_lig_•••HN_prot_, and N_lig_•••HN_prot_) were additionally analyzed according to homogenous substitutions with only Fluorine; Chlorine, Bromine or Iodine. All heterogeneously substituted ligands (e.g. bromo-fluoro or chloro-iodo) were excluded from this analysis.

Multiple protein molecules in the crystal cell, as well as objects displaying partially occupied forms (i.e. side-chain rotamers or ligand locations) were analyzed separately. Hydrogen bonds with water molecules were not analyzed.

### Statistical Analysis

To circumvent the eventual requirement of categorization, all distributions are presented in a cumulative manner as a CDF (cumulative distribution function), which is the integral of a distribution function. This form of presentation helps in visual comparison of various distributions, overcoming the problem of balancing the resolution (i.e. the number of beans in a histogram) and statistical noise (i.e. numbers of counts in beans). In all figures the curve most shifted to the left identifies the dataset characterized by the shortest donor-acceptor distances.

Since, according to the Anderson-Darling test [65], most distributions of hydrogen bond donor-acceptor distances were found not Gaussian (data not shown), the statistical significance of observed differences was estimated according to nonparametric tests. For comparison of two distributions the Mann-Whitney U test [66] was used. The Kruskal-Wallis test [67], which is a generalization of the U-test, was applied for 3 or more groups.

The Mann-Whitney U-test is a first choice alternative to Student’s t-test, when applied to two data-sets that are not necessarily normally distributed. Formally, it detects differences in shape of tested distributions: each group is characterized by its mean rank, i.e. the average position of its components in the list created by sorting both datasets. For each pair of distributions, the smaller value of the mean rank (R_i_) identifies the group that is characterized by a shorter distance (see Tables 2, 3). The value of U_i_ is the corresponding test statistics, (U_i_ = n_i*_[R_i_−(n_i_+1)/2]; n_i_ is the size of dataset *i*), and Z_U_ is the associated value of the standard Gaussian distribution. Positive Z_U_ value (equivalently higher mean rank for LH) indicates that distances for halogenated ligands are shorter, and the corresponding p-value estimates the statistical significance of observed differences. The medians were also compared for selected pairs of distributions according to the appropriate median test [68].

All analyses were performed using the Statistica 10 [69]. Null hypotheses that given distributions do not differ one from the other were tested at a significance level, α = 0.05, and those with p-values below 0.05 were rejected, and distributions regarded as different.

## Conclusions

Hydrogen bond length distributions in protein-ligand complexes are significantly different for non-halogenated ligands (LH) compared to halogenated ones (LF, LX). The H-bond donor-acceptor distances are significantly shorter for a halogenated ligand acting as a hydrogen bond donor (at significance level 0.05). However H-bond lengths seem irrelevant for halogenations, when the ligand oxygen is a hydrogen bond acceptor. All these observations are consistent with the idea that halogenation increases the acidity of proximal amino/imino/hydroxyl groups and thus makes them better, i.e. stronger, H-bond donors.

## Supporting Information

Figure S1 Cumulative distributions of donor-acceptor distances determined for various types of intermolecular hydrogen bond donor-acceptor pairs identified In complexes of proteins with non-halogenated ligands, in which the ligand is either a hydrogen bond donor (A, C, E) or acceptor (B, D, F); determined for non-halogenated, LH, (A, B), fluorinated, LF, (C, D), and otherwise halogenated (i.e. not fluorinated), LX, ligands (E, F).
(TIF)

Figure S2 Effect of a halogen atom on cumulative distributions determined for the donor-acceptor pairs determined for hydrogen bonds between ligand and protein backbone (carbonyl oxygen: A, C or amide nitrogen” B, D). The distributions estimated for non-halogenated, fluorinated and otherwise halogenated ligands are presented in black, blue and red, respectively.
(TIF)

Table S1 Results of the Kruskal-Wallis (K-W) test in the analysis of the topology-dependent length of a hydrogen bond: for each pair of hydrogen bond acceptor/donor pair the p-value for the null hypothesis that both distributions are identical was estimated according to the two-tailed multiple comparison. The values marked in bold denote the pairs of distributions that differ one from the other, with α = 0.05. Additionally, the identified number of each type of hydrogen bond, n, and mean rank test are presented.
(DOC)

Table S2 Comparison of distributions of hydrogen bond lengths, calculated separately for halogenated but not fluorinated (LX), fluorinated (LF), and non-halogenated ligands (LH), for eight possible topologies of protein-ligand hydrogen bonds. Those for which hydrogen bonds to LX/LF ligands are, according to the Mann-Whitney U test, significantly shorter (assuming α = 0.05) are highlighted. Note that for each pair of H-bond distributions, a smaller mean rank indicates statistically shorter donor-acceptor distances, or, equivalently, positive values of Z_U_ statistics indicate these types of H-bonds, which are longer to nonhalogenated ligands. The corresponding medians, and their differences with statistical significances (p), are also presented.
(DOC)

Table S3 Comparison of distributions of hydrogen bond lengths, calculated separately for ligands fluorinated (LF), otherwise halogenated (LX), and non-halogenated (LH), for hydrogen bonds between ligand and protein that were identified in high-resolution X-ray structures.
(DOC)
