# Supplementary figures and images for: A Protein Data Bank Survey Reveals Shortening of Intermolecular Hydrogen Bonds in Ligand-Protein Complexes When a Halogenated Ligand Is an H-Bond Donor
Source: PLoS One. 2014 Jun 16;9(6):e99984. doi: 10.1371/journal.pone.0099984 (PMC4059718; doi:10.1371/journal.pone.0099984)

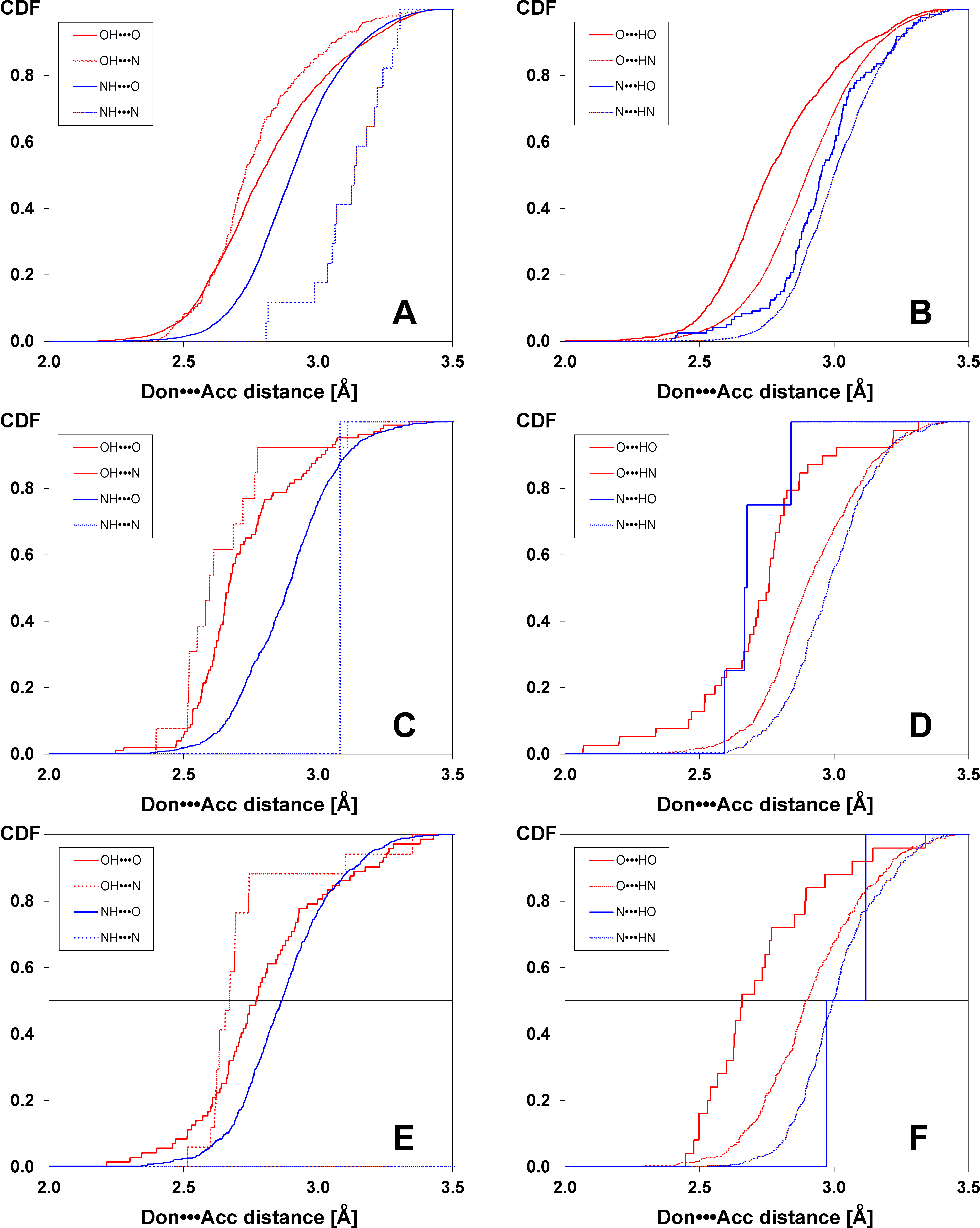

Supplement: Figure S1 — Cumulative distributions of donor-acceptor distances determined for various types of intermolecular hydrogen bond donor-acceptor pairs identified In complexes of proteins with non-halogenated ligands, in which the ligand is either a hydrogen bond donor (A, C, E) or acceptor (B, D, F); determined for non-halogenated, LH, (A, B), fluorinated, LF, (C, D), and otherwise halogenated (i.e. not fluorinated), LX, ligands (E, F). (TIF) [file pone.0099984.s001.tif]

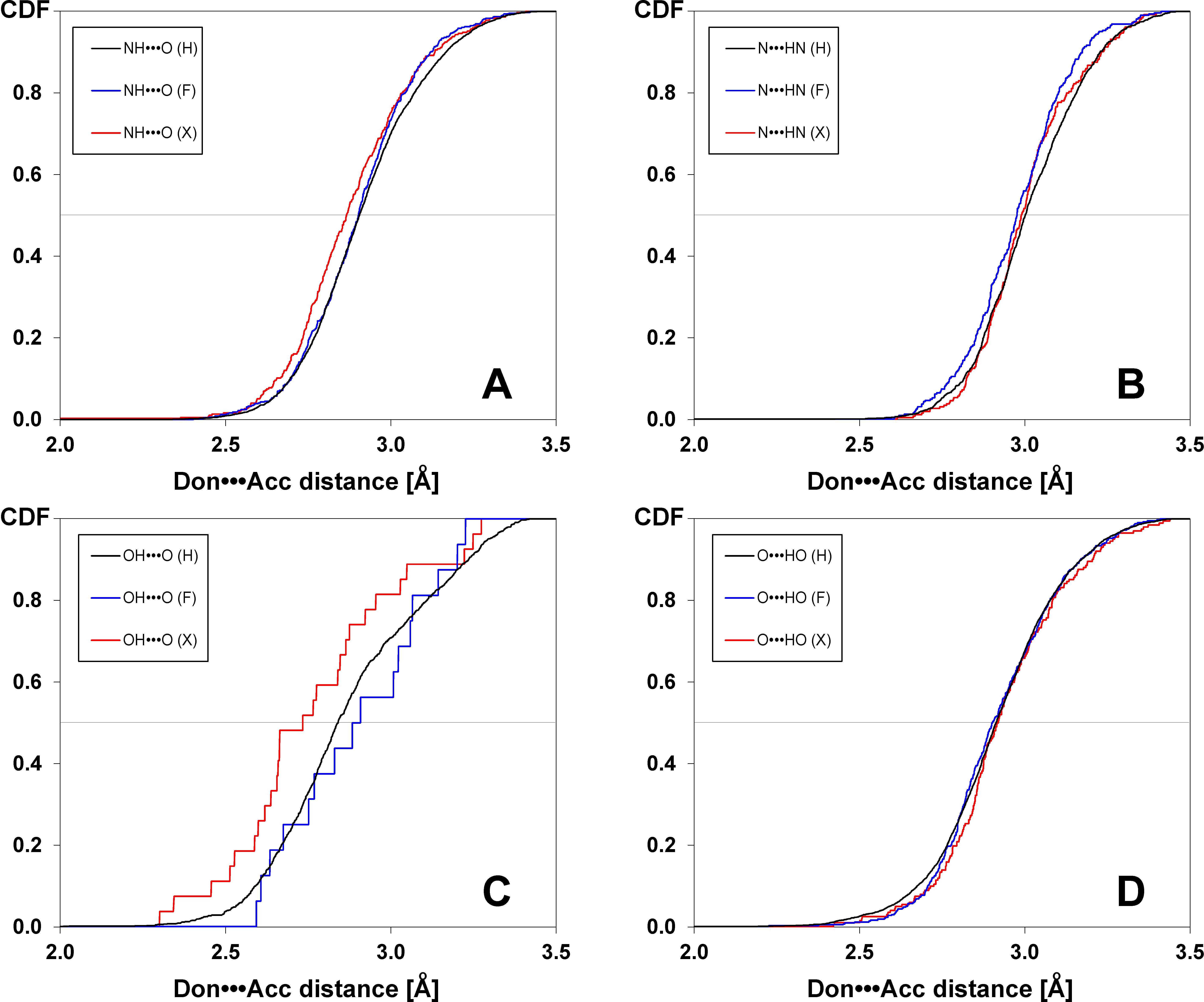

Supplement: Figure S2 — Effect of a halogen atom on cumulative distributions determined for the donor-acceptor pairs determined for hydrogen bonds between ligand and protein backbone (carbonyl oxygen: A, C or amide nitrogen” B, D). The distributions estimated for non-halogenated, fluorinated and otherwise halogenated ligands are presented in black, blue and red, respectively. (TIF) [file pone.0099984.s002.tif]
