# Supplementary material for: A Protein Data Bank Survey Reveals Shortening of Intermolecular Hydrogen Bonds in Ligand-Protein Complexes When a Halogenated Ligand Is an H-Bond Donor
Source: PLoS One. 2014 Jun 16;9(6):e99984. doi: 10.1371/journal.pone.0099984 (PMC4059718; doi:10.1371/journal.pone.0099984)
Supplement: Table S1 — Results of the Kruskal-Wallis (K-W) test in the analysis of the topology-dependent length of a hydrogen bond: for each pair of hydrogen bond acceptor/donor pair the p-value for the null hypothesis that both distributions are identical was estimated according to the two-tailed multiple comparison. The values marked in bold denote the pairs of distributions that differ one from the other, with α = 0.05. Additionally, the identified number of each type of hydrogen bond, n, and mean rank test are presented. (DOC) [file pone.0099984.s003.doc]

| **H-bond**  **topology** | n | Mean rank | H-bond topology (ligand ∙ protein) | | | | | | | |
| --- | --- | --- | --- | --- | --- | --- | --- | --- | --- | --- |
|  |  |  | OH∙∙∙O | OH∙∙∙N | NH∙∙∙O | NH∙∙∙N | O∙∙∙HO | O∙∙∙HN | N∙∙∙HO | N∙∙∙HN |
| **LH: K-W test for non-halogenated ligands: H(7, N= 21150) =1519; p < 10^-9^** | | | | | | | | | | |
| **OH∙∙∙O** | **3358** | **8378** | - | **1.9·10^-3^** | **<1·10^-10^** | **1.6·10^-7^** | **7.6·10^-3^** | **<1·10^-10^** | **<1·10^-10^** | **<1·10^-10^** |
| OH∙∙∙N | 251 | 6787 | **1.9·10^-3^** | - | **<1·10^-10^** | **6.2·10^-10^** | 1 | **<1·10^-10^** | **<1·10^-10^** | **<1·10^-10^** |
| **NH∙∙∙O** | **5670** | **11015** | **<1·10^-10^** | **<1·10^-10^** | - | **1.4·10^-3^** | **<1·10^-10^** | 1 | **0.05** | **<1·10^-10^** |
| NH∙∙∙N | 17 | 17026 | **1.6·10^-7^** | **6.2·10^-10^** | **1.4·10^-3^** | - | **9.1·10^-9^** | **1.1·10^-3^** | 0.20 | 1 |
| O∙∙∙HO | 1331 | 7659 | **7.6·10^-3^** | 1 | **<1·10^-10^** | **9.1·10^-9^** | - | **<1·10^-10^** | **<1·10^-10^** | **<1·10^-10^** |
| **O∙∙∙HN** | **8675** | **10943** | **<1·10^-10^** | **<1·10^-10^** | 1 | **1.1·10^-3^** | **<1·10^-10^** | - | **0.03** | **<1·10^-10^** |
| N∙∙∙HO | 121 | 12769 | **<1·10^-10^** | **<1·10^-10^** | **0.05** | 0.20 | **<1·10^-10^** | **0.03** | - | 0.47 |
| **N∙∙∙HN** | **1727** | **14140** | **<1·10^-10^** | **<1·10^-10^** | **<1·10^-10^** | 1 | **<1·10^-10^** | **<1·10^-10^** | 0.47 | - |
| **LF: K-W test for fluorinated ligands: H(7, N= 1930) =212; p < 10^-9^** | | | | | | | | | | |
| **OH∙∙∙O** | **103** | **471** | - | 1 | **<1·10^-10^** | 1 | 1 | **<1·10^-10^** | 1 | **<1·10^-10^** |
| OH∙∙∙N | 13 | 294 | 1 | - | **1.5·10^-3^** | 0**.**64 | 1 | **1.2·10^-4^** | 1 | **1.3·10^-7^** |
| **NH∙∙∙O** | **842** | **922** | **<1·10^-10^** | **1.5·10^-3^** | - | 1 | **4.0·10^-4^** | 0.09 | 0.91 | **<1·10^-10^** |
| NH∙∙∙N | 1 | 1612 | 1 | 0**.**64 | 1 | - | 1 | 1 | 1 | 1 |
| O∙∙∙HO | 39 | 526 | 1 | 1 | **4.0·10^-4^** | 1 | - | **4.0·10^-6^** | 1 | **<1·10^-10^** |
| **O∙∙∙HN** | **571** | **1011** | **<1·10^-10^** | **1.2·10^-4^** | 0.09 | 1 | **4.0·10^-6^** | - | 0.40 | **1.6·10^-6^** |
| N∙∙∙HO | 4 | 325 | 1 | 1 | 0.91 | 1 | 1 | 0.40 | - | 0.04 |
| **N∙∙∙HN** | **357** | **1215** | **<1·10^-10^** | **1.3·10^-7^** | **<1·10^-10^** | 1 | **<1·10^-10^** | **1.6·10^-6^** | 0.04 | - |
| **LX: K-W test for ligands halogenated, not fluorinated: H(6, N= 1390) =171; p<10^-9^** | | | | | | | | | | |
| **OH∙∙∙O** | **72** | **489** | - | 1 | 0**.**11 |  | 1 | **3.3·10^-4^** | 1 | **<1·10^-10^** |
| OH∙∙∙N | 17 | 278 | 1 | - | **7.9·10^-3^** |  | 1 | **2.5·10^-4^** | 0.22 | **2.6·10^-9^** |
| **NH∙∙∙O** | **659** | **629** | 0**.**11 | **7.9·10^-3^** | - |  | **0.02** | **0.03** | 1 | **<1·10^-10^** |
| NH∙∙∙N | 0 |  |  |  |  |  |  |  |  |  |
| O∙∙∙HO | 25 | 355 | 1 | 1 | **0.02** |  | - | **3.2·10^-4^** | 0.41 | **2.4·10^-10^** |
| **O∙∙∙HN** | **317** | **716** | **3.3·10^-4^** | **2.5·10^-4^** | **0.03** |  | **3.2·10^-4^** | - | 1 | **3.9·10^-9^** |
| N∙∙∙HO | 2 | 1044 | 1 | 0.22 | 1 |  | 0.41 | 1 | - | 1 |
| **N∙∙∙HN** | **298** | **922** | **<1·10^-10^** | **2.6·10^-9^** | **<1·10^-10^** |  | **2.4·10^-10^** | **3.9·10^-9^** | 1 | - |
