# Supplementary material for: A Protein Data Bank Survey Reveals Shortening of Intermolecular Hydrogen Bonds in Ligand-Protein Complexes When a Halogenated Ligand Is an H-Bond Donor
Source: PLoS One. 2014 Jun 16;9(6):e99984. doi: 10.1371/journal.pone.0099984 (PMC4059718; doi:10.1371/journal.pone.0099984)
Supplement: Table S2 — Comparison of distributions of hydrogen bond lengths, calculated separately for halogenated but not fluorinated (LX), fluorinated (LF), and non-halogenated ligands (LH), for eight possible topologies of protein-ligand hydrogen bonds. Those for which hydrogen bonds to LX/LF ligands are, according to the Mann-Whitney U test, significantly shorter (assuming α = 0.05) are highlighted. Note that for each pair of H-bond distributions, a smaller mean rank indicates statistically shorter donor-acceptor distances, or, equivalently, positive values of ZU statistics indicate these types of H-bonds, which are longer to nonhalogenated ligands. The corresponding medians, and their differences with statistical significances (p), are also presented. (DOC) [file pone.0099984.s004.doc]

| **H-bond**  **topology** | **n** | | **Mean rank** | | **U statistics** | **Z_U_** | **p-value** | **Median [Å]** | | | **p-value** |
| --- | --- | --- | --- | --- | --- | --- | --- | --- | --- | --- | --- |
|  | **LH** | **LF** | **LH** | **LF** |  |  |  | **LH** | **LF** | **Δ(LF-LH)** |  |
| **OH∙∙∙O** | 3358 | 103 | 1743.9 | 1309.5 | 129525 | **4.35** | **1.4·10^-5^** | **2.787** | **2.668** | **-0.119** | **7.2·10^-6^** |
| **OH∙∙∙N** | 251 | 13 | 135.1 | 81.6 | 970 | **2.46** | **1.4·10^-2^** | **2.728** | **2.597** | **-0.131** | **0.05** |
| **NH∙∙∙O** | 5670 | 842 | 3286.9 | 3051.9 | 2214771 | **3.38** | **7.1·10^-4^** | 2.899 | 2.885 | -0.014 | 0.18 |
| NH∙∙∙N | 17 | 1 |  |  |  |  |  | 3.135 | 3.081 | -0.054 | 0.31 |
| O∙∙∙HO | 1331 | 39 | 687.7 | 611.1 | 23052 | 1.19 | 0.23 | 2.757 | 2.758 | 0.001 | 0.86 |
| O∙∙∙HN | 8675 | 571 | 4616.4 | 4731.6 | 2414967 | -1.00 | 0.32 | 2.900 | 2.895 | -0.005 | 0.76 |
| **N∙∙∙HO** | 121 | 4 | 64.6 | 14.8 | 49 | **2.70** | **6.9·10^-3^** | **2.953** | **2.671** | **-0.282** | **0.04** |
| **N∙∙∙HN** | 1727 | 357 | 1060.4 | 956.0 | 277400 | **2.98** | **2.9·10^-3^** | **3.001** | **2.976** | **-0.025** | **0.03** |
|  | **LH** | **LX** | **LH** | **LX** |  |  |  | **LH** | **LX** | **Δ(LX-LH)** |  |
| OH∙∙∙O | 3358 | 72 | 1717.0 | 1645.5 | 115847 | 0.61 | 0.54 | 2.787 | 2.769 | -0.018 | 0.35 |
| **OH∙∙∙N** | 251 | 17 | 136.5 | 104.3 | 1621 | 1.66 | 0.10 | **2.728** | **2.667** | **-0.061** | **0.02** |
| **NH∙∙∙O** | 5670 | 659 | 3201.7 | 2849.2 | 1660152 | **4.69** | **2.8·10^-6^** | **2.899** | **2.864** | **-0.035** | **4.9·10^-5^** |
| NH∙∙∙N | 17 | 0 |  |  |  |  |  | 3.135 |  |  |  |
| O∙∙∙HO | 1331 | 25 | 680.9 | 550.7 | 13442 | 1.65 | 0.10 | 2.757 | 2.657 | -0.100 | 0.16 |
| O∙∙∙HN | 8675 | 317 | 4492.7 | 4601.6 | 1341680 | -0.73 | 0.46 | 2.900 | 2.897 | -0.003 | 0.69 |
| N∙∙∙HO | 121 | 2 | 61.7 | 83.0 | 79 | -0.83 | 0.41 | 2.953 | 3.045 | 0.092 | 0.15 |
| N∙∙∙HN | 1727 | 298 | 1014.2 | 1006.0 | 255250 | 0.22 | 0.82 | 3.001 | 2.998 | -0.003 | 0.81 |
