# Supplementary material for: A Protein Data Bank Survey Reveals Shortening of Intermolecular Hydrogen Bonds in Ligand-Protein Complexes When a Halogenated Ligand Is an H-Bond Donor
Source: PLoS One. 2014 Jun 16;9(6):e99984. doi: 10.1371/journal.pone.0099984 (PMC4059718; doi:10.1371/journal.pone.0099984)
Supplement: Table S3 — Comparison of distributions of hydrogen bond lengths, calculated separately for ligands fluorinated (LF), otherwise halogenated (LX), and non-halogenated (LH), for hydrogen bonds between ligand and protein that were identified in high-resolution X-ray structures. (DOC) [file pone.0099984.s005.doc]

**For structures with resolution better than 2.0Å**

| **H-bond**  **topology** | **n** | | **Mean rank** | | **U statistics** | **Z_U_** | **p-value** | **Median [Å]** | | | **p-value** |
| --- | --- | --- | --- | --- | --- | --- | --- | --- | --- | --- | --- |
|  | **LH** | **LF** | **LH** | **LF** |  |  |  | **LH** | **LF** | **Δ(LF-LH)** |  |
| OH∙∙∙O | 1286 | 32 | 669.5 | 402.5 | 12352 | 6.05 | 1.5·10^-9^ | 2.773 | 2.655 | -0.118 | 1.6·10^-6^ |
| NH∙∙∙O | 2000 | 274 | 1144.7 | 1085.1 | 259642 | 1.41 | 0.16 | 2.904 | 2.902 | -0.002 | 0.91 |
| O∙∙∙HN | 3038 | 167 | 1598.5 | 1685.1 | 240002 | -1.17 | 0.24 | 2.899 | 2.892 | -0.007 | 0.83 |
| N∙∙∙HN | 377 | 71 | 232.0 | 184.8 | 10565 | 2.83 | 4.7·10^-3^ | 3.036 | 2.969 | -0.067 | 1.4·10^-2^ |
|  | **LH** | **LX** | **LH** | **LX** |  |  |  | **LH** | **LX** | **Δ(LX-LH)** |  |
| OH∙∙∙O | 1286 | 20 | 654.4 | 594.5 | 11680 | 0.69 | 0.49 | 2.773 | 2.735 | -0.038 | 0.18 |
| NH∙∙∙O | 2000 | 277 | 1156.3 | 1014.0 | 242375 | 3.37 | 7.4·10^-4^ | 2.904 | 2.866 | -0.038 | 4.2·10^-5^ |
| O∙∙∙HN | 3038 | 96 | 1568.8 | 1525.9 | 141830 | 0.45 | 0.65 | 2.899 | 2.892 | -0.007 | 0.70 |
| N∙∙∙HN | 377 | 69 | 227.2 | 203.5 | 11627 | 1.42 | 0.16 | 3.036 | 2.992 | -0.044 | 0.03 |

**For structures with resolution better than 1.5Å**

| **H-bond**  **topology** | **n** | | **Mean rank** | | **U statistics** | **Z_U_** | **p-value** | **Median [Å]** | | | **p-value** |
| --- | --- | --- | --- | --- | --- | --- | --- | --- | --- | --- | --- |
|  | **LH** | **LF** | **LH** | **LF** |  |  |  | **LH** | **LF** | **Δ(LF-LH)** |  |
| OH∙∙∙O | 274 | 5 | 141.3 | 68.6 | 328 | 1.99 | 4.6·10^-2^ | 2.729 | 2.622 | -0.107 | 2.5·10^-2^ |
| NH∙∙∙O | 388 | 29 | 212.1 | 167.4 | 4421 | 1.92 | 5.4·10^-2^ | 2.938 | 2.902 | -0.035 | 3.8·10^-2^ |
| O∙∙∙HN | 658 | 20 | 340.8 | 295.6 | 5701 | 1.02 | 0.31 | 2.923 | 2.881 | -0.043 | 0.36 |
| N∙∙∙HN | 23 | 8 | 17.6 | 11.5 | 56 | 1.60 | 0.11 | 3.068 | 2.954 | -0.114 | 0.12 |
|  | **LH** | **LX** | **LH** | **LX** |  |  |  | **LH** | **LX** | **Δ(LX-LH)** |  |
| OH∙∙∙O | 274 | 1 |  |  |  |  |  | 2.729 | 2.683 | -0.046 | 0.32 |
| NH∙∙∙O | 388 | 74 | 243.2 | 170.1 | 9813 | 4.32 | 1.6·10^-5^ | 2.938 | 2.844 | -0.094 | 1.6·10^-5^ |
| O∙∙∙HN | 658 | 10 | 334.9 | 306.2 | 3007 | 0.47 | 0.64 | 2.923 | 2.917 | -0.006 | 0.99 |
| N∙∙∙HN | 23 | 8 | 15.7 | 16.8 | 86 | -0.25 | 0.80 | 3.068 | 3.079 | 0.011 | 0.92 |

**Supplementary Figure 1**. Cumulative distributions of donor-acceptor distances determined for various types of intermolecular hydrogen bond donor-acceptor pairs identified in complexes of proteins with non-halogenated ligands, in which the ligand is either a hydrogen bond donor (A, C, E) or acceptor (B, D, F); determined for non-halogenated, LH, (A, B), fluorinated, LF, (C, D), and halogenated (not fluorinated), LX, ligands (E, F).

**Supplementary Figure 2.** Effect of a halogen atom on cumulative distributions determined for the donor-acceptor pairs determined for hydrogen bonds between ligand and protein backbone (carbonyl oxygen: A,C or amide nitrogen” B,D). The distributions estimated for non-halogenated, fluorinated and halogenated ligands are presented in black, blue and red, respectively.
